# Impacto da Obesidade em Pacientes com Hipertensão Arterial Resistente e Refratária: Um Estudo Transversal

**DOI:** 10.36660/abc.20250627

**Published:** 2026-02-26

**Authors:** Bárbara Victória Peixoto Lima da Costa, Fábio Bulhões, Cristiano Macedo, Alex Cleber Improta-Caria, Roque Aras

**Affiliations:** 1 Faculdade Medicina da Bahia Universidade Federal da Bahia Salvador BA Brasil Faculdade Medicina da Bahia - Universidade Federal da Bahia (FMB-UFBA), Salvador, BA – Brasil; 2 Programa de Pós-Graduação em Medicina e Saúde Universidade Federal da Bahia Salvador BA Brasil Programa de Pós-Graduação em Medicina e Saúde (PPGMS), Universidade Federal da Bahia, Salvador, BA – Brasil; 3 Laboratório de Bioquímica e Biologia Molecular do Exercício Universidade de São Paulo São Paulo SP Brasil Laboratório de Bioquímica e Biologia Molecular do Exercício - Universidade de São Paulo (USP), São Paulo, SP – Brasil

**Keywords:** Obesidade, Prevalência, Hipertensão

## Abstract

**Fundamento:**

A obesidade é um grave problema de saúde pública e está associada à ativação do sistema nervoso simpático e ao desenvolvimento de hipertensão resistente ou refratária.

**Objetivos:**

Estimar a prevalência de obesidade na população de pacientes ambulatoriais com hipertensão refratária ou resistente e sua relação entre índice de massa corporal e gravidade da hipertensão.

**Métodos:**

Foi realizado um estudo transversal em um ambulatório de referência para hipertensão arterial grave. Foram incluídos pacientes com hipertensão resistente e refratária. Foram coletadas informações relacionadas às características demográficas, medidas antropométricas, pressão arterial sistólica e diastólica, comorbidades, alcoolismo, tabagismo e sedentarismo. As variáveis contínuas foram comparadas utilizando o teste t de Student ou Mann-Whitney, e as variáveis categóricas foram comparadas pelo teste do qui-quadrado. O teste de correlação de Pearson foi utilizado para medidas antropométricas e pressão arterial. Foi adotado nível de significância de p < 0,05.

**Resultados:**

Um total de 138 pacientes foi incluído, dos quais 74,7% apresentavam hipertensão resistente e 25,3% hipertensão refratária. Houve predominância de mulheres e de indivíduos de etnia negra, representando 79,7% (p = 0,307) e 91,3% (p = 0,315), respectivamente. A média de idade foi de 64,7 ± 10,8 anos (p = 0,566). A obesidade (IMC ≥ 30 kg/m^2^) esteve presente em 42% (p = 0,379), sendo mais frequente na hipertensão refratária (51,4% vs. 38,8%). Valores mais elevados de IMC estiveram diretamente associados a um maior número de medicamentos anti-hipertensivos necessários para o controle da pressão arterial (r = 0,45; p < 0,01). Além disso, 74,6% dos pacientes apresentavam dislipidemia.

**Conclusão:**

Em nossa população de pacientes com hipertensão grave, a obesidade foi prevalente e impactou tanto na apresentação quanto no controle dos níveis de pressão arterial.

## Graphical abstract

**Figura Central:**
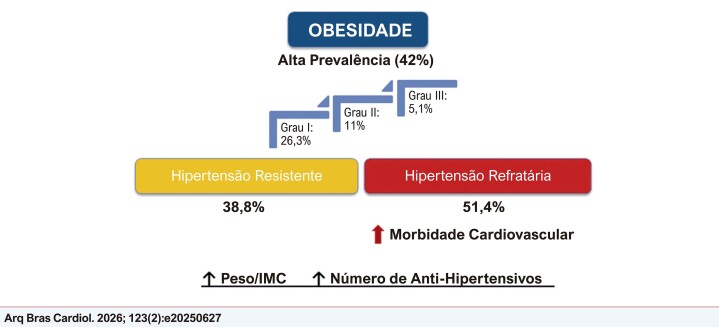
Impacto da Obesidade em Pacientes com Hipertensão Arterial Resistente e Refratária: Um Estudo Transversal

Diferentes níveis de obesidade contribuem para o desenvolvimento de hipertensão resistente ou refratária, tornando necessário adaptar o uso de medicamentos anti-hipertensivos de acordo com o grau de obesidade. IMC: índice de massa corporal.

## Introdução

A obesidade é definida pela Organização Mundial da Saúde como um acúmulo excessivo de gordura, com índice de massa corporal (IMC) maior ou igual a 30 Kg/m^2^.^1^Esse distúrbio multifatorial tem sido reconhecido como um grave problema de saúde pública global devido à sua alta prevalência, aos estilos de vida sedentários e ao amplo consumo de alimentos ultraprocessados e hipercalóricos.^2^ Em 2015, 603,7 milhões de pessoas em todo o mundo foram afetadas, e a Associação Médica Americana classificou a obesidade como uma doença crônica.^3 , 4^

A obesidade é considerada um fator de risco significativo para o desenvolvimento de doenças cardiovasculares, apneia do sono, diabetes mellitus tipo 2, síndrome metabólica e hipertensão arterial.^5 , 6^ Assim, contribui para o processo aterosclerótico ao prejudicar a sensibilidade à insulina e promover um estado inflamatório arterial, que induz a disfunção endotelial,^5^ além de aumentar o perfil inflamatório no coração e em diversos órgãos.

A obesidade está envolvida na ativação do sistema nervoso simpático e está associada ao desenvolvimento de hipertensão resistente ou refratária.^7^ A hipertensão resistente (HR) refere-se a pacientes cuja pressão arterial (PA) permanece não controlada (≥ 140/90 mmHg), apesar do uso de três ou mais classes de medicamentos, sendo um deles obrigatoriamente um diurético tiazídico; ou àqueles cuja PA só é controlada com quatro ou mais fármacos. Já a hipertensão refratária (HRf) corresponde a uma forma mais grave, envolvendo pacientes que não conseguem controlar os níveis de PA mesmo com cinco ou mais medicamentos anti-hipertensivos, incluindo a espironolactona.^8^

Pacientes com HRf apresentam maior prevalência de lesão em órgãos-alvo quando comparados àqueles com HR, sendo mais propensos a apresentar taxa de filtração glomerular reduzida, albuminúria, diabetes, acidente vascular cerebral (AVC) e doença arterial coronariana (DAC).^8 - 10^ Além disso, estudos demonstraram que indivíduos com IMC superior a 40 têm sete vezes mais chances de serem hipertensos em comparação com indivíduos eutróficos.^2^

No entanto, poucos estudos avaliaram a prevalência da obesidade na HRf. Assim, o objetivo deste estudo foi analisar o impacto e a prevalência da obesidade em uma população de pacientes com HR ou HRf e a relação entre IMC e controle da PA.

## Métodos

### Delineamento do estudo

Este é um estudo descritivo, transversal, com abordagem quantitativa e descritiva, realizado em um ambulatório de referência para hipertensão arterial grave em um hospital público de atenção terciária.

### População do estudo

A amostra foi selecionada por conveniência, composta por pacientes em tratamento de HR e HRf. Por se tratar de uma amostra de conveniência, não foi realizado cálculo formal do tamanho da amostra. Todos os pacientes elegíveis foram incluídos consecutivamente durante consultas de acompanhamento de rotina no ambulatório de hipertensão grave, no período de junho de 2018 a março de 2020. Os dados foram coletados por meio de entrevistas e revisão de prontuários médicos. A PA foi medida com esfigmomanômetro aneroide em ambos os membros superiores, com o paciente sentado, após cinco minutos de repouso, de acordo com a técnica padronizada descrita pelas Diretrizes Americanas.^11^

Os valores registrados referem-se à média das medidas obtidas em ambos os braços. Foram incluídos pacientes com HR – definida como PA maior ou igual a 140/90 mmHg durante o uso de três ou mais classes de medicamentos anti-hipertensivos nas doses máximas recomendadas ou toleradas, incluindo um diurético tiazídico; ou pacientes com PA controlada (<140/90 mmHg) que necessitavam de quatro ou mais medicamentos. Também foram incluídos pacientes com HRf – aqueles com PA não controlada (≥140/90 mmHg), apesar do uso de cinco ou mais fármacos anti-hipertensivos, incluindo espironolactona e um diurético de longa ação.^8^

O peso foi aferido utilizando uma balança calibrada, e a altura, por meio de um estadiômetro. O IMC foi calculado dividindo-se o peso do paciente (Kg) pelo quadrado da altura (m^2^). A obesidade foi definida como IMC ≥30 Kg/m^2^, o sobrepeso como IMC ≥25 Kg/m^2^, e o peso normal como IMC entre 18,5 e 24,9 Kg/m^2^. A obesidade foi classificada em três categorias: obesidade grau 1 (IMC 30,0–34,9 Kg/m^2^), obesidade grau 2 (IMC 35,0–39,9 Kg/m^2^) e obesidade grau 3 (IMC ≥40 Kg/m^2^).

A circunferência abdominal foi medida utilizando uma fita métrica posicionada no ponto médio entre a borda inferior da última costela e a crista ilíaca. A obesidade abdominal foi definida como circunferência da cintura maior que 94 cm em homens ou maior que 80 cm em mulheres.^12^ A relação cintura/altura (RCA) também foi calculada dividindo-se a circunferência da cintura pela altura. Os dados coletados incluíram idade, sexo, altura, peso, pressão arterial sistólica (PAS), pressão arterial diastólica (PAD), pressão arterial média, circunferência abdominal, níveis de creatinina, presença de diabetes mellitus, dislipidemia, relatos de insuficiência cardíaca congestiva (ICC), DAC, AVC, depressão, medicamentos em uso, consumo de álcool, tabagismo e inatividade física.

### Análise estatística

Os dados foram inseridos em uma planilha do Microsoft Excel^®^ 2016 e analisados no software Jamovi^®^. As variáveis contínuas foram apresentadas como média ± desvio padrão, e as variáveis categóricas como frequência absoluta e percentual relativo.

Para avaliar a distribuição das variáveis, foram utilizadas estatísticas descritivas, análise gráfica, assimetria, curtose e o teste de normalidade (Shapiro-Wilk). Para comparar os grupos HR e HRf, aplicou-se o teste t de Student ou o teste de Mann-Whitney, de acordo com a distribuição das variáveis quantitativas, e o teste do qui-quadrado para variáveis categóricas.

O teste de correlação de Pearson foi aplicado para analisar as correlações entre variáveis antropométricas e de PA. Foi adotado nível de significância de p < 0,05.

Todos os participantes assinaram o termo de consentimento livre e esclarecido para participação no estudo. O projeto foi aprovado pelo Comitê de Ética em Pesquisa do Hospital Universitário Professor Edgard Santos (HUPES/UFBA), sob o protocolo nº 81701717.6.0000.0049.

## Resultados

Um total de 138 pacientes foi analisado, dos quais 74,7% apresentavam HR e 25,3% HRf. Houve predominância de indivíduos idosos (≥60 anos), do sexo feminino e de pacientes de ascendência africana em ambos os grupos, conforme detalhado na Tabela 1 . A idade média geral foi 64,7 anos. Esse padrão reflete as características demográficas da população local atendida em nosso ambulatório terciário, que é conhecido por ter maior proporção de mulheres idosas e indivíduos de ascendência africana, em consonância com as características epidemiológicas locais e os dados de saúde pública. A prevalência de obesidade (IMC ≥30 Kg/m^2^) na amostra foi de 42,0%, com Intervalo de Confiança (IC) de 95% entre 33,8% e 50,2%.

**Tabela 1:** – Características demográficas e clínicas dos pacientes

Características	Total 138 (100%)	Hipertensão Resistente 103 (74.7%)	Hipertensão refratária 35 (25.3%)	Valor p
Sexo feminino	110 (79,7%)	80 (77,7%)	30 (85,7%)	0,307
Etnia negra	126 (91,3%)	91 (88,3%)	35 (100%)	0,315
Idade ≥ 60 anos	97 (70,3%)	74 (71,8%)	23 (65,7%)	0,493
Idade (média)	64,7 ± 10,8	65 ± 11,1	63,8 ± 10	0,566
IMC (Kg/m^2^)	29,8 ± 6,1	28,6 (25,6-32,8)	30,1 (27,4-33,3)	0,194
PAS (mmHg)	152,4 ± 30,6	150 (139-168)	154 (142-170)	0,342
PAD (mmHg)	85,3 ± 17,3	84 (76,3-93)	88,5 (80,3-99,8)	0,059
Circunferência da cintura (cm)	98,5 ± 15,3	97,4 ± 15,7	102 ± 13,5	0,141
Relação Cintura/Altura (RCA)	0,63 ± 0,1	0,6 (0,6-0,7)	0,7 (0,6-0,7)	0,177
Creatinina (mg/dL)	1,01 ± 0,3	0,9 (0,8-1,1)	0,9 (0,8-1,3)	0,120
Diabetes Mellitus	71 (51,4%)	51 (49,5%)	20 (57,1%)	0,435
Síndrome metabólica	98 (71%)	72 (69,9%)	26 (74,3%)	0,581
Dislipidemia	103 (74,6%)	74 (71,8%)	29 (82,9%)	0,400
ICC prévia	23 (16,7%)	15 (14,6%)	8 (22,9%)	0,389
DAC prévia	38 (27,5%)	23 (22,3%)	15 (42,9%)	**0,019**
AVC prévio	24 (17,4%)	14 (13,6%)	10 (28,6%)	**0,043**
Depressão	30 (21,7%)	22 (21,4%)	8 (22,9%)	0,853
Obesidade	58 (42%)	40 (38,8%)	18 (51,4%)	0,379
Consumo de álcool	26 (18,8%)	21 (20,4%)	5 (14,3%)	0,425
História de tabagismo	38 (27,7%)	28 (27,2%)	10 (28,6%)	0,836
Tabagismo atual	5 (3,6%)	3 (2,9%)	2 (5,7%)	0,443
Inatividade física	67 (48,6%)	49 (47,6%)	18 (51,4%)	0,693

*IMC: índice de massa corporal; PAS: pressão arterial sistólica; PAD: pressão arterial diastólica; RCA: relação cintura/altura; ICC: insuficiência cardíaca congestiva; DAC: doença arterial coronariana; AVC: acidente vascular cerebral.*

Em relação ao IMC, a média geral foi de 29,8 ± 6,1 Kg/m^2^, com os pacientes com HRf apresentando valores numericamente mais elevados. A circunferência abdominal média também foi numericamente maior entre os pacientes com HRf em comparação aos com HR, e a RCA seguiu tendência semelhante. Os valores detalhados estão apresentados na Tabela 1 . A PAS média foi de 152,4 ± 30,6 mmHg, com valores semelhantes entre os grupos. A PAD média foi de 85,3 ± 17,3 mmHg, numericamente mais elevada no grupo HRf.

Em relação à presença de comorbidades, 16,7% apresentavam ICC, 17,4% tinham histórico de AVC, 21,7% apresentavam depressão, 27,5% tinham DAC, 51,4% eram portadores de diabetes mellitus, 71% apresentavam síndrome metabólica e 74,6% dislipidemia. Não houve diferença estatisticamente significativa entre os grupos em relação ao diabetes mellitus, síndrome metabólica, dislipidemia, insuficiência cardíaca e depressão. No entanto, a DAC e o AVC prévio foram mais prevalentes no grupo HRf (42,9% vs 22,3% e 28,6% vs 13,6%, respectivamente). Os níveis de creatinina foram ligeiramente mais elevados nos pacientes com HRf em comparação aos pacientes com HR – mediana de 0,9 mg/dL (IQR: 0,8-1,3) versus 0,9 mg/dL (IQR: 0,8–1,1), respectivamente (p = 0,120), conforme mostrado na Tabela 1 .

Em relação aos fatores de estilo de vida, a inatividade física foi proeminente, afetando quase metade da amostra (48,6%). O consumo de álcool apresentou prevalência de 18,8%, e o tabagismo de 27,7%, embora apenas 3,6% dos participantes fossem fumantes atuais. Entre os indivíduos com HR e HRf, a prevalência de obesidade foi de 38,8% e 51,4%, respectivamente. Na distribuição geral das categorias de IMC, 2,2% dos participantes estavam abaixo do peso, 19% tinham peso normal, 36,5% apresentavam sobrepeso, 26,3% obesidade grau I, 11% obesidade grau II e 5,1% obesidade grau III.

**Figura 1:**
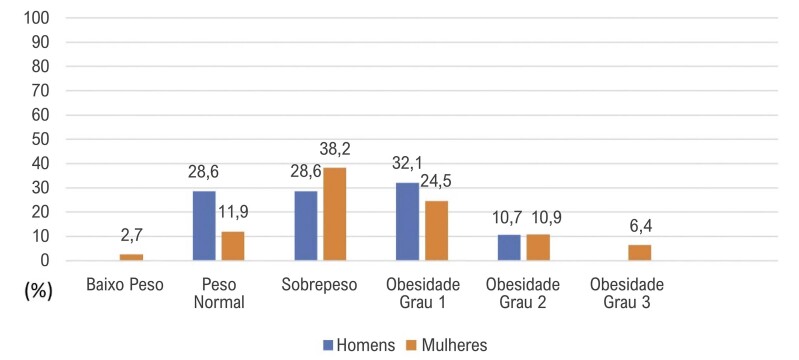
– Distribuição das categorias de índice de massa corporal por sexo.

Ao estratificar as categorias de IMC por sexo, verificou-se que entre os homens, 28,6% tinham peso normal, 28,6% apresentavam sobrepeso, 32,1% obesidade grau I e 10,7% obesidade grau II. Entre as mulheres, 2,7% estavam abaixo do peso, 11,9% tinham peso normal, 38,2% apresentavam sobrepeso, 24,5% foram classificadas com obesidade grau I, 10,9% com obesidade grau II e 6,4% com obesidade grau III ( Figura 1 ).

Uma análise da média do IMC em relação ao número de agentes anti-hipertensivos utilizados revelou uma tendência linear e progressiva. Pacientes em uso de três medicamentos apresentaram média de IMC de 28,2; aqueles em uso de quatro medicamentos tiveram média de 30,4; cinco medicamentos corresponderam a média de 30,6; seis medicamentos a média de 30,1; sete medicamentos a média de 30,5; e oito medicamentos a média de 35,5. Esses valores estão resumidos na Tabela 2 .

**Tabela 2:** – Estatísticas descritivas – número de medicamentos anti-hipertensivos em relação ao Índice de Massa Corporal (IMC), distribuídos por grupos de hipertensão

Variáveis	HR (3 medicamentos)	HR (4 medicamentos)	HRf (5 medicamentos)	HRf (6 medicamentos)	HRf (7 medicamentos)	HRf (8 medicamentos)
n (pacientes)	40	63	25	7	1	2
IMC (Kg/m^2^), mean ± SD	28,2 ± 5,8	30,4 ± 6,2	30,7 ± 4,5	30,1 ± 10,9	30,5	35,5 ± 8,8

*HR: hipertensão resistente; HRf: hipertensão resistente ou refratária. Para sete medicamentos, somente um paciente foi incluído (não se aplica o desvio padrão).*

**Figura 2:**
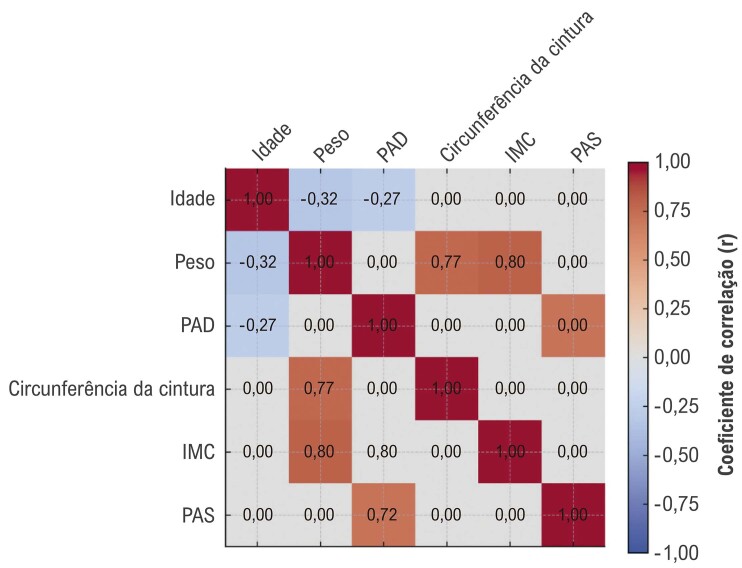
– Mapa de calor da correlação de Pearson entre medidas antropométricas e variáveis de pressão arterial; IMC: índice de massa corporal; PAS: pressão arterial sistólica; PAD: pressão arterial diastólica.

Além disso, o teste de correlação de Pearson foi empregado para avaliar as correlações entre as variáveis, incluindo sua significância estatística e proporcionalidade ( Tabela 3 e Figura 2 ). As correlações entre idade e peso, idade e PAD, peso e circunferência abdominal, IMC e circunferência abdominal, PAS e PAD foram estatisticamente significativas. Todas as correlações foram diretamente proporcionais, exceto as correlações entre idade e peso, e idade e PAD, que foram inversamente proporcionais.

**Tabela 3:** – Correlação de Pearson entre medidas antropométricas e pressão arterial

Pares de variáveis	Coeficiente de correlação (r)	Valor p
Idade × Peso	-0,323	< 0,001
Idade × PAD	-0,268	0,001
Peso × Circunferência da cintura	0,766	< 0,001
IMC × Circunferência da cintura	0,798	< 0,001
PAS × PAD	0,716	< 0,001

*IMC: índice de massa corporal; PAS: pressão arterial sistólica; PAD: pressão arterial diastólica.

## Discussão

A obesidade é um importante fator de risco e contribui para complicações nas doenças cardiovasculares. Em nossa população de pacientes com hipertensão grave, a obesidade foi prevalente e impactou na apresentação e no controle dos níveis de pressão arterial. A literatura mostra que indivíduos obesos têm risco significativamente maior de desenvolver hipertensão de difícil controle.^5 , 6 , 13^ Isso se deve a mecanismos fisiopatológicos como ativação do sistema nervoso simpático, retenção de sódio e resistência insulínica, que exacerbam o processo aterosclerótico.^5^ No presente estudo, a prevalência de obesidade, considerando todos os indivíduos com IMC maior ou igual a 30 Kg/m^2^, foi de 42%, confirmando a associação previamente observada em outros estudos que indicam que pacientes com IMC elevado têm maior probabilidade de desenvolver HR ou HRf.^2^

Nossos resultados mostram uma distribuição estratificada entre as diferentes categorias de gravidade da obesidade, medida pelo IMC. Dos pacientes, 26,3% foram classificados como obesidade grau 1, 11% como obesidade grau 2 e 5,1% como obesidade grau 3, reforçando essa relação, consistente com o estudo de Vildoso et al.,^14^ que observaram em uma população de pacientes com HR um IMC médio de 31,01 ± 5,60 Kg/m^2^, com alterações significativas no perfil lipídico.^1^

Observamos uma predominância de mulheres (80%), com níveis mais elevados de pressão arterial sistólica e IMC em comparação aos homens. Também vale destacar a maior idade dos participantes; 71% tinham 60 anos ou mais, o que, nas mulheres, sugere que a menopausa pode influenciar a relação entre hipertensão e obesidade por meio da redução dos níveis de estrogênio, levando à perda da proteção cardiovascular, aumento da adiposidade e resistência insulínica.^15^ Em consonância com nossos resultados, Sun et al.^16^ relataram, em uma coorte de 156 624 mulheres americanas pós-menopausa, que a obesidade central, mesmo em indivíduos com peso normal, esteve associada a maior risco de mortalidade por doenças cardiovasculares, sugerindo a influência da gordura visceral nesses eventos.

De forma semelhante, Ferreira-Campos et al.^17^ relataram que mulheres em uso de terapia de reposição hormonal apresentaram menores chances de hipertensão (OR=0,59; IC95%: 0,41–0,85) em comparação àquelas que nunca a utilizaram.^17^ Em relação à cor da pele, é importante destacar que os afrodescendentes foram prevalentes em nossa população (91%), refletindo as características étnicas da cidade e do estado onde o estudo foi conduzido, sugerindo maior gravidade na apresentação da hipertensão.^18 , 19^ Essa abordagem é particularmente relevante porque os dados sobre obesidade, HR e HRf em populações afrodescendentes no Brasil são escassos.^10^ A maioria dos estudos sobre HR foi realizada em populações predominantemente caucasianas, limitando a generalização de seus achados para o contexto brasileiro. Ao analisar esse grupo sub-representado, nosso estudo fornece uma visão sobre a prevalência e o impacto da obesidade em pacientes afrodescendentes.

É importante destacar que a média da RCA foi de 0,63 ± 0,1, acima do valor de corte de 0,5, indicando alto risco de desenvolvimento de doenças cardiovasculares ou metabólicas. Os níveis de creatinina e as medidas antropométricas, incluindo IMC e circunferência abdominal, foram numericamente maiores em pacientes com HRf em comparação àqueles com HR, embora as diferenças não tenham sido estatisticamente significativas. O estudo de Modolo et al.^20^ relatou valores de creatinina de 1,15 ± 0,73 *vs* . 1,01 ± 0,41 e IMC de 32,1 ± 5,6 vs 31,9 ± 6,9, destacando a presença de obesidade na maioria das amostras.

Além disso, o aumento do IMC foi diretamente proporcional ao número de medicamentos anti-hipertensivos utilizados, conforme mostrado na Tabela 2 , indicando que pacientes obesos tendem a necessitar de mais fármacos para o controle adequado da pressão arterial. Esse achado é consistente com o estudo de Cataldi et al.,^21^ que discute a eficácia limitada da monoterapia em pacientes com hipertensão relacionada à obesidade. Kotchen^13^ explora ainda a relação entre obesidade e hipertensão, observando que 60-70% dos casos de hipertensão em adultos podem estar associados ao excesso de adiposidade, resistência insulínica e disfunção endotelial. Ele também enfatiza que o manejo clínico de pacientes obesos hipertensos deve incluir modificações no estilo de vida. Por outro lado, Park et al.^22^ destacam a existência da pseudo-HR, que pode estar relacionada à hipertensão do avental branco, subdosagem de medicamentos, baixa adesão ao tratamento e medidas imprecisas da pressão arterial no consultório.

Além da obesidade, observamos alta prevalência de doenças cardiovasculares ( Figura Central ), como insuficiência cardíaca (16,7%), AVC prévio (17,4%), DAC (27,5%), bem como diabetes mellitus (51,4%), síndrome metabólica (71%), dislipidemia (74,6%) e depressão (21,7%). Esses achados estão em concordância com o estudo de Sim et al.,^23^ que comparou indivíduos sem HR com aqueles com HR e verificou que o grupo com HR apresentava maior prevalência de comorbidades, incluindo diabetes mellitus (48% vs 30%), doença renal crônica (45% vs 24%), doença cardíaca isquêmica (41% vs 22%) e doença cerebrovascular (16% vs 9%).

A dislipidemia foi a comorbidade mais prevalente em nosso estudo; de forma semelhante, Wallace et al.^24^ demonstraram uma associação direta entre os níveis séricos de LDL-C e maior incidência de doenças cardiovasculares. Esses achados reforçam a importância do controle do colesterol LDL na prevenção de complicações cardiovasculares, especialmente em pacientes hipertensos, nos quais a dislipidemia representa um fator de risco significativo que deve ser abordado como parte do manejo clínico.^24^

Além disso, as comorbidades foram mais prevalentes no grupo com HRf em comparação ao grupo com HR, conforme observado por Modolo et al.,^20^ que relataram que a obesidade afetava 61% dos hipertensos refratários e 55% dos hipertensos resistentes. De forma semelhante, Cardoso e Salles^27^ identificaram que o diagnóstico de HRf estava associado a riscos significativamente maiores de desfechos adversos, mortalidade cardiovascular e incidência de AVC em comparação aos pacientes com HR.

Esses achados enfatizam a importância da adesão adequada à medicação, combinada a estratégias voltadas para a perda de peso em pacientes com HR.^25^ Uma meta-análise de 25 ensaios clínicos randomizados demonstrou que a redução da pressão arterial associada à perda de peso foi proporcional.^28^ Além da redução da pressão arterial, sugere-se que a perda de peso também diminua o dano a órgãos-alvo, reduza a excreção urinária de albumina e promova a regressão da hipertrofia ventricular esquerda.^10 , 20 , 27 , 29 - 31^

Finalmente, nosso estudo apresenta algumas limitações que devem ser consideradas na interpretação dos resultados. Em primeiro lugar, seu desenho transversal impede o estabelecimento de relações causais entre obesidade e controle da hipertensão, restringindo os achados apenas a associações. Além disso, o uso de uma amostra de conveniência proveniente de um ambulatório de atenção terciária limita a generalização dos resultados para outras populações e contextos de saúde, especialmente aqueles com diferentes perfis demográficos ou clínicos. A avaliação da obesidade foi baseada principalmente no IMC, que não distingue entre massa de gordura e massa muscular magra. Métodos mais precisos, como a análise de bioimpedância, poderiam fornecer uma melhor compreensão da composição corporal e de sua relação com o controle da pressão arterial. Para mitigar essa limitação, incluímos outras medidas antropométricas — como circunferência abdominal e RCA – que refletem a adiposidade central e estão mais estreitamente relacionadas ao risco cardiovascular.

Apesar dessas limitações, o estudo oferece informações valiosas sobre os perfis antropométricos e clínicos de pacientes com hipertensão grave, destacando a complexidade do manejo da pressão arterial em indivíduos obesos. Vale ressaltar também que este trabalho é um dos poucos estudos que avaliam a prevalência e o impacto da obesidade na HRf e HR em uma população brasileira, com predominância de pacientes afrodescendentes e mulheres acima de 60 anos. Essas características tornam os achados relevantes para a compreensão de grupos vulneráveis que frequentemente estão sub-representados na pesquisa clínica.

## Conclusão

Em nossa população de pacientes com hipertensão grave, a obesidade foi prevalente e impactou na apresentação e no controle dos níveis de pressão arterial. O aumento do IMC foi diretamente proporcional ao uso de múltiplos medicamentos anti-hipertensivos, indicando que pacientes obesos tendem a necessitar de mais fármacos para o controle adequado da pressão arterial.
