# Supplementary material for: Impacto da Obesidade em Pacientes com Hipertensão Arterial Resistente e Refratária: Um Estudo Transversal
Source: Arq Bras Cardiol. 2026 Feb 26;123(2):e20250627. [Article in Portuguese] doi: 10.36660/abc.20250627 (PMC13128177; doi:10.36660/abc.20250627)
Supplement: Translation

# Translation: Impact of Obesity on Patients with Resistant and Refractory Arterial Hypertension: A Cross-Sectional Study

**DOI:** 10.36660/abc.20250627i

**Authors:** Bárbara Victória Peixoto Lima da Costa, Fábio Bulhões, Cristiano Macedo, Alex Cleber Improta-Caria, Roque Aras

**Affiliations:** 1 Brasil Faculdade Medicina da Bahia - Universidade Federal da Bahia (FMB-UFBA), Salvador, BA – Brasil; 2 Brasil Programa de Pós-Graduação em Medicina e Saúde (PPGMS), Universidade Federal da Bahia, Salvador, BA – Brasil; 3 Brasil Laboratório de Bioquímica e Biologia Molecular do Exercício - Universidade de São Paulo (USP), São Paulo, SP – Brasil

**Keywords:** Obesity, Prevalence, Hypertension

## Abstract

**Background:**

Obesity is a serious public health problem and is associated with activation of the sympathetic nervous system and the development of resistant or refractory hypertension.

**Objectives:**

To estimate the prevalence of obesity in the population of outpatients with refractory or resistant hypertension and its relationship between body mass index and hypertension severity.

**Methods:**

A cross-sectional study was carried out in a referral outpatient clinic for severe arterial hypertension. Patients with resistant and refractory hypertension were included. Information related to demographic characteristics, anthropometric measurements, systolic and diastolic blood pressure, comorbidities, alcoholism, smoking, and sedentary lifestyle was collected. Continuous variables were compared using the Student’s t-test or Mann Whitney, and categorical variables were compared using the chi-square test. Pearson’s correlation test was used for anthropometric measurements and blood pressure. A significance level of p < 0.05 was adopted.

**Results:**

A total of 138 patients were collected, of whom 74.7% had resistant hypertension and 25.3% had refractory hypertension. There was a predominance of females and black ethnicities, representing 79.7% (p=0.307) and 91.3% (p=0.315), respectively. The mean age was 64.7± 10.8 (p=0.566). Obesity (BMI ≥ 30 kg/m^2^) was present in 42% (p=0.379), being higher in refractory hypertension (51.4% vs. 38.8%). Higher body mass index values were directly associated with an increased number of antihypertensive medications required for blood pressure control (r = 0.45; p < 0.01). Additionally, 74.6% of the patients had dyslipidemia (p=0.400).

**Conclusion:**

In our population of patients with severe hypertension, obesity was prevalent and significantly influenced both the presentation and the control of blood pressure levels.

## Introduction

Obesity is defined by the World Health Organization as an excessive accumulation of fat, with a body mass index (BMI) greater than or equal to 30 Kg/m^2^.^1^This multifactorial disorder has been addressed as a serious public health issue on a global scale, considering its high prevalence, sedentary lifestyle, and the consumption of ultra-processed and high-calorie foods.^2^In 2015, 603.7 million people worldwide were affected, and it has been classified as a chronic disease by the American Medical Association.^3 , 4^

Obesity is considered a significant risk factor for the development of cardiovascular diseases, sleep apnea, type 2 diabetes mellitus, metabolic syndrome, and arterial hypertension.^5 , 6^Thus, it contributes to the atherosclerotic process by impairing insulin sensitivity and promoting an arterial inflammatory state, which induces endothelial dysfunction,^5^in addition to increasing the inflammatory profile in the heart and various organs.

Obesity is involved in the activation of the sympathetic nervous system and is associated with the development of resistant or refractory hypertension.^7^Resistant hypertension (RH) refers to patients whose blood pressure (BP) remains uncontrolled (≥ 140/90 mmHg), despite the use of three or more classes of drugs, one of which must be a thiazide diuretic; or those whose BP is controlled only with four or more medications. Refractory hypertension (RfH), on the other hand, refers to a more severe form, involving patients who are unable to control BP levels even with five or more antihypertensive drugs, including spironolactone.^8^

Patients with RfH show a higher prevalence of target organ damage when compared to those with RH, being more likely to present with lower glomerular filtration rate, albuminuria, diabetes, stroke, and coronary artery disease (CAD).^8 - 10^Moreover, studies have shown that individuals with a BMI greater than 40 are seven times more likely to be hypertensive when compared to eutrophic individuals.^2^

However, few studies have assessed the prevalence of obesity in RfH. Thus, the aim of this study was to evaluate the impact and prevalence of obesity in a population of patients with RH or RfH and the relationship between BMI and BP control.

## Methods

### Study design

This is a descriptive, cross-sectional study with a quantitative and descriptive approach, conducted at a referral outpatient clinic for severe arterial hypertension in a public tertiary care hospital.

### Study population

The sample was selected by convenience, consisting of patients being treated for RH and RfH. As this was a convenience sample, no formal sample size calculation was performed. All eligible patients were consecutively included during routine follow-up visits at the severe hypertension outpatient clinic from June 2018 to March 2020. Data were collected through interviews and medical record reviews. BP was measured using an aneroid sphygmomanometer on both the right and left upper limbs, with the patient seated, after five minutes of rest, according to the standardized technique outlined by the American Guidelines.^11^

The recorded values refer to the average of the measurements taken from both arms. Patients with RH were included – defined as BP greater than or equal to 140/90 mmHg while on three or more classes of antihypertensive medications at the maximum recommended or tolerated doses, including a thiazide diuretic, or patients with controlled BP (<140/90 mmHg) requiring four or more medications. Patients with RfH were also included – those with uncontrolled BP (≥140/90 mmHg) despite taking five or more antihypertensive drugs, including spironolactone and a long-acting diuretic.^8^

Weight was measured using a calibrated scale, and height using a stadiometer. BMI was calculated by dividing the patient’s weight (Kg) by the square of their height (m^2^). Obesity was defined as a BMI ≥30 Kg/m^2^, overweight as a BMI ≥25 Kg/m^2^, and normal weight as a BMI between 18.5 and 24.9 Kg/m^2^. Obesity was classified into three categories: Grade 1 obesity (BMI 30.0–34.9 Kg/m^2^), Grade 2 obesity (BMI 35.0–39.9 kg/m^2^), and Grade 3 obesity (BMI ≥40 Kg/m^2^).

Abdominal circumference was measured using a measuring tape placed at the midpoint between the lower edge of the last rib and the iliac crest. Abdominal obesity was defined as a waist circumference greater than 94 cm in men or greater than 80 cm in women.^12^The waist-to-height ratio (WHtR) was also calculated by dividing waist circumference by height. Data collected included age, sex, height, weight, systolic blood pressure (SBP), diastolic blood pressure (DBP), mean arterial pressure, abdominal circumference, creatinine levels, presence of diabetes mellitus, dyslipidemia, reports of congestive heart failure (CHF), CAD, stroke, depression, medications in use, alcohol use, smoking, and physical inactivity.

### Statistical analysis

The data were entered into a Microsoft Excel^®^2016 spreadsheet and analyzed in the Jamovi^®^software. Continuous variables were presented as mean ± standard deviation, and categorical variables as absolute frequency and relative percentage.

To evaluate the distribution of the variables, descriptive statistics, graphical analysis, asymmetry, kurtosis, and the normality test (Shapiro-Wilk) were used. To compare the HR and HRf groups, the Student’s t-test or the Mann-Whitney test were applied, according to the distribution of quantitative variables, and the chi-square test for categorical variables.

Pearson’s correlation test was applied to analyze the correlations between anthropometric and blood pressure variables. A significance level of p < 0.05 was adopted.

All participants signed the informed consent form to participate in the study. The project was approved by the Research Ethics Committee of Professor Edgard Santos University Hospital (HUPES/UFBA), under protocol number 81701717.6.0000.0049.

## Results

A total of 138 patients were analyzed, of whom 74.7% had RH and 25.3% had RfH. There was a predominance of elderly individuals (≥60 years), females, and patients of African descent in both groups, as detailed in Table 1 . The overall mean age was 64.7 years. This pattern reflects the demographic characteristics of the local population served by our tertiary outpatient clinic, which is known to have a higher proportion of elderly women and individuals of African descent, consistent with local epidemiological characteristics and public health data. The prevalence of obesity (BMI ≥30 kg/m^2^) in the sample was 42.0%, with a 95% confidence interval (CI) of 33.8% to 50.2%.

**Table 1:** – Demographic and clinical characteristics

Characteristics	Total 138 (100%)	Resistant Hypertension 103 (74.7%)	Refractory Hypertension 35 (25.3%)	p-value
Female sex	110 (79.7%)	80 (77.7%)	30 (85.7%)	0.307
Black ethnicity	126 (91.3%)	91 (88.3%)	35 (100%)	0.315
Age ≥ 60 years	97 (70.3%)	74 (71.8%)	23 (65.7%)	0.493
Age (mean)	64.7 ± 10.8	65 ± 11.1	63.8 ± 10	0.566
BMI (kg/m^2^)	29.8 ± 6.1	28.6 (25.6-32.8)	30.1 (27.4-33.3)	0.194
SBP (mmHg)	152.4 ± 30.6	150 (139-168)	154 (142-170)	0.342
DBP (mmHg)	85.3 ± 17.3	84 (76.3-93)	88.5 (80.3-99.8)	0.059
Waist circumference (cm)	98.5 ± 15.3	97.4 ± 15.7	102 ± 13.5	0.141
Waist-to-height ratio (WHtR)	0.63 ± 0.1	0.6 (0.6-0.7)	0.7 (0.6-0.7)	0.177
Creatinine (mg/dL)	1.01 ± 0.3	0.9 (0.8-1.1)	0.9 (0.8-1.3)	0.120
Diabetes Mellitus	71 (51.4%)	51 (49.5%)	20 (57.1%)	0.435
Metabolic syndrome	98 (71%)	72 (69.9%)	26 (74.3%)	0.581
Dyslipidemia	103 (74.6%)	74 (71.8%)	29 (82.9%)	0.400
Prior CHF	23 (16.7%)	15 (14.6%)	8 (22.9%)	0.389
Prior CAD	38 (27.5%)	23 (22.3%)	15 (42.9%)	**0.019**
Prior stroke	24 (17.4%)	14 (13.6%)	10 (28.6%)	**0.043**
Depression	30 (21.7%)	22 (21.4%)	8 (22.9%)	0.853
Obesity	58 (42%)	40 (38.8%)	18 (51.4%)	0.379
Alcohol use	26 (18.8%)	21 (20.4%)	5 (14.3%)	0.425
Smoking history	38 (27.7%)	28 (27.2%)	10 (28.6%)	0.836
Current smoking	5 (3.6%)	3 (2.9%)	2 (5.7%)	0.443
Physical inactivity	67 (48.6%)	49 (47.6%)	18 (51.4%)	0.693

*BMI: body mass index; SBP: systolic blood pressure; DBP: diastolic blood pressure; WHtR: waist-to-height ratio; CHF: congestive heart failure; CAD: coronary artery disease.*

Regarding BMI, the overall average was 29.8 ± 6.1 Kg/m^2^, with RfH patients showing numerically higher values. The average waist circumference was also numerically higher among RfH patients than RH patients, and the WHtR followed a similar trend. Detailed values are shown in Table 1 . The mean SBP was 152.4 ± 30.6 mmHg, with similar values between the groups. The mean DBP was 85.3 ± 17.3 mmHg, numerically higher in the RfH group.

Regarding the presence of comorbidities, 16.7% had CHF, 17.4% had a previous stroke, 21.7% had depression, 27.5% had CAD, 51.4% had diabetes mellitus, 71% had metabolic syndrome, and 74.6% had dyslipidemia. There was no statistically significant difference between the groups in relation to diabetes mellitus, metabolic syndrome, dyslipidemia, heart failure, and depression. However, CAD and prior stroke were more prevalent in the RfH group (42.9% vs 22.3% and 28.6% vs 13.6%, respectively). Creatinine levels were slightly higher in patients with RfH compared to patients with RH — median of 0.9 mg/dL (IQR: 0.8–1.3) versus 0.9 mg/dL (IQR: 0.8–1.1), respectively (p = 0.120), as shown in Table 1 .

Regarding lifestyle factors, physical inactivity was prominent, affecting nearly half of the sample (48.6%). Alcohol consumption had a prevalence of 18.8%, and smoking 27.7%, although only 3.6% of participants were current smokers. Among individuals with RH and RfH, the prevalence of obesity was 38.8% and 51.4%, respectively. In the overall distribution of BMI categories, 2.2% of participants were underweight, 19% had normal weight, 36.5% were overweight, 26.3% had class I obesity, 11% had class II obesity, and 5.1% had class III obesity.

Stratifying BMI categories by sex revealed that among males, 28.6% had normal weight, 28.6% were overweight, 32.1% presented with class I obesity, and 10.7% with class II obesity. Among females, 2.7% were underweight, 11.9% had normal weight, 38.2% were overweight, 24.5% were classified as having class I obesity, 10.9% as class II obesity, and 6.4% as class III obesity ( Figure 1 ).

**Figure 1:** – Distribution of body mass index categories by gender.

An analysis of the mean BMI in relation to the number of antihypertensive agents used revealed a linear and progressive trend. Patients using three medications had a mean BMI of 28.2; those on four medications had a mean BMI of 30.4; five medications corresponded to a mean BMI of 30.6; six medications to a mean BMI of 30.1; seven medications to a mean BMI of 30.5; and eight medications to a mean BMI of 35.5. These values are summarized in Table 2 .

**Table 2:** – Descriptive Statistics – Number of antihypertensive medications in relation to body mass index (BMI), distributed by hypertension groups

Variables	RH (3 drugs)	RH (4 drugs)	RfH (5 drugs)	RfH (6 drugs)	RfH (7 drugs)	RfH (8 drugs)
n (patients)	40	63	25	7	1	2
BMI (kg/m^2^), mean ± SD	28.2 ± 5.8	30.4 ± 6.2	30.7 ± 4.5	30.1 ± 10.9	30.5	35.5 ± 8.8

RH: resistant hypertension; RfH: refractory hypertension. For 7 medications, only one patient was included — standard deviation not applicable.

Furthermore, Pearson’s Correlation Test was employed to assess the correlations between variables, including their statistical significance and proportionality ( Table 3 and Figure 2 ). The correlations between age and weight, age and DBP, weight and waist circumference, BMI and waist circumference, SBP and DBP were statistically significant. All correlations were directly proportional, except for the correlations between age and weight, and age and DBP, which were inversely proportional.

**Table 3:** – Pearson correlation between anthropometric measurements and blood pressure

Variable Pair	Correlation Coefficient (r)	p-value
Age × Weight	-0.323	< 0.001
Age × DBP	-0.268	0.001
Weight × Waist circumference	0.766	< 0.001
BMI × Waist circumference	0.798	< 0.001
SBP × DBP	0.716	< 0.001

*BMI: body mass index; SBP: systolic blood pressure; DBP: diastolic blood pressure.

**Figure 2:** – Pearson correlation heatmap between anthropometric measurements and blood pressure variables. BMI: body mass index; SBP: systolic blood pressure; DBP: diastolic blood pressure.

## Discussion

Obesity is an important risk factor and contributor to complications in cardiovascular diseases. In our population of patients with severe hypertension, obesity was prevalent and impacted on the presentation and control of BP levels. The literature shows that obese individuals have a significantly higher risk of developing difficult-to-control hypertension.^5 , 6 , 13^This is due to pathophysiological mechanisms such as sympathetic nervous system activation, sodium retention, and insulin resistance, which exacerbate the atherosclerotic process.^5^In the present study, the prevalence of obesity, considering all individuals with a BMI greater than or equal to 30 Kg/m^2^, was 42%, confirming the association previously observed in other studies that indicate patients with elevated BMI are more likely to develop RH or RfH.^2^

Our results show a stratified distribution among the different obesity severity categories measured by BMI. 26.3% were classified as having grade 1 obesity, 11% with grade 2 obesity, and 5.1% with grade 3 obesity, reinforcing this relationship, which is consistent with the study by Vildoso et al.,^14^who observed in a population of patients with RH an average BMI of 31.01 ± 5.60 kg/m^2^, with significant alterations in lipid profile.^1^

We observed a predominance of females (80%), with higher levels of systolic blood pressure and BMI compared to males. It is also worth highlighting the older age of participants; 71% were aged 60 years or older, which in women suggests that menopause may influence the relationship between hypertension and obesity through a reduction in estrogen levels, leading to loss of cardiovascular protection, increased adiposity, and insulin resistance.^15^Consistent with our results, Sun et al.^16^reported in a cohort of 156,624 postmenopausal American women that central obesity, even in individuals with normal weight, was associated with an increased risk of mortality from cardiovascular diseases, suggesting the influence of visceral fat on these events.

Similarly, Ferreira-Campos et al.^17^reported that women using hormone replacement therapy had lower odds of hypertension (OR=0.59; 95% CI: 0.41–0.85) compared to those who never used it.^17^Regarding skin color, it is important to note that Afro-descendants were prevalent in our population (91%), reflecting the ethnic characteristics of the city and state where the study was conducted, suggesting greater severity in the presentation of hypertension.^18 , 19^This approach is particularly relevant because data on obesity, RH and RfH in Afro-descendant populations in Brazil are scarce.^10^Most studies on RH have been conducted in predominantly Caucasian populations, limiting the generalization of their findings to Brazilian environments. By analyzing this underrepresented group, our study provides insight into the prevalence and impact of obesity in patients of African descent.

It is noteworthy that the mean WHR was 0.63 ± 0.1, which is above the cutoff value of 0.5, indicating a high risk of developing cardiovascular or metabolic diseases. Creatinine levels and anthropometric measures, including BMI and abdominal circumference, were numerically higher in patients with RfH compared to those with RH, although the differences were not statistically significant. The study by Modolo et al.^20^reported creatinine values of 1.15 ± 0.73 vs 1.01 ± 0.41 and BMI of 32.1 ± 5.6 vs 31.9 ± 6.9, highlighting the presence of obesity in most of the samples.

Furthermore, the increase in BMI was directly proportional to the number of antihypertensive drugs used, as shown in Table 2 , indicating that obese patients tend to require more medications for adequate BP control. This finding is consistent with the study by Cataldi et al.,^21^which discusses the limited effectiveness of monotherapy in patients with obesity-related hypertension. Kotchen^13^further explores the relationship between obesity and hypertension, noting that 60–70% of adult hypertension cases may be associated with excess adiposity, insulin resistance, and endothelial dysfunction. He also emphasizes that the clinical management of obese hypertensive patients should include lifestyle modifications. Conversely, Park et al.^20^highlight the existence of pseudo-RH, which may result from white coat hypertension, suboptimal dosing, poor medication adherence, and inaccurate office blood pressure measurements.

In addition to obesity, we observed a high prevalence of cardiovascular diseases ( Central Illustration ), such as heart failure (16.7%), previous stroke (17.4%), CAD (27.5%), as well as diabetes mellitus (51.4%), metabolic syndrome (71%), dyslipidemia (74.6%), and depression (21.7%). These findings agree with the study by Sim et al.,^23^which compared individuals without RH to those with RH and found that the RH group had a higher prevalence of comorbidities, including diabetes mellitus (48% vs 30%), chronic kidney disease (45% vs 24%), ischemic heart disease (41% vs 22%), and cerebrovascular disease (16% vs 9%).

Dyslipidemia was the most prevalent comorbidity in our study; similarly, Wallace et al.^24^demonstrated a direct association between serum LDL-C levels and a higher incidence of cardiovascular diseases. These findings reinforce the importance of LDL cholesterol control in preventing cardiovascular complications, especially in hypertensive patients, where dyslipidemia represents a significant risk factor that must be addressed as part of clinical management.^24^

Furthermore, comorbidities were more prevalent in the RfH group compared to the RH group, as observed by Modolo et al.,^20^who reported that obesity affected 61% of refractory hypertensives and 55% of resistant hypertensive patients. Similarly, Cardoso and Salles^27^identified that a diagnosis of RfH was associated with significantly higher risks of adverse outcomes, cardiovascular mortality, and stroke incidence compared to RH patients.

These findings emphasize the importance of proper medication adherence combined with strategies aimed at weight loss in patients with RH.^25^A meta-analysis of 25 randomized clinical trials demonstrated that the reduction in blood pressure associated with weight loss was proportional.^28^Beyond blood pressure reduction, weight loss has also been suggested to decrease target organ damage, reduce urinary albumin excretion, and promote regression of left ventricular hypertrophy.^10 , 20 , 27 , 29 - 31^

Finally, our study presents some limitations that should be considered when interpreting the results. First, its cross-sectional design prevents establishing causal relationships between obesity and hypertension control, restricting the findings to associations only. Additionally, the use of a convenience sample from a tertiary care outpatient clinic limits the generalizability of the results to other populations and healthcare settings, especially those with different demographic or clinical profiles. The assessment of obesity was primarily based on BMI, which does not differentiate between fat mass and lean muscle mass. More precise methods, such as bioimpedance analysis, could provide a better understanding of body composition and its relationship with blood pressure control. To mitigate this, we included other anthropometric measures – such as waist circumference and WHR – that reflect central adiposity and are more closely linked to cardiovascular risk.

Despite these limitations, the study offers valuable insights into the anthropometric and clinical profiles of patients with severe hypertension, highlighting the complexity of managing blood pressure in obese individuals. It is also worth noting that this work is one of the few studies that evaluate the prevalence and impact of obesity on RfH and RH in a Brazilian population, with a predominance of Afro-descendant patients and women over 60 years of age. These characteristics make the findings relevant to understanding vulnerable groups that are often underrepresented in clinical research.

## Conclusion

In our population of patients with severe hypertension, obesity was prevalent and impacted on the presentation and control of BP levels. Increase in BMI was directly proportional to the use of multiple antihypertensive medications, indicating that obese patients tend to require more drugs for adequate BP control.
